# FlightTrackAI: a robust convolutional neural network-based tool for tracking the flight behaviour of *Aedes aegypti* mosquitoes

**DOI:** 10.1098/rsos.240923

**Published:** 2024-10-02

**Authors:** Nouman Javed, Adam J. López-Denman, Prasad N. Paradkar, Asim Bhatti

**Affiliations:** ^1^ Institute for Intelligent Systems Research and Innovation, Deakin University, Geelong, Victoria 3216, Australia; ^2^ CSIRO Health & Biosecurity, Australian Centre for Disease Preparedness, Geelong, Victoria 3220, Australia

**Keywords:** disease transmission, convolutional neural network, multi-object tracking, flight behaviour, FlightTrackAI, vector control

## Abstract

Monitoring the flight behaviour of mosquitoes is crucial for assessing their fitness levels and understanding their potential role in disease transmission. Existing methods for tracking mosquito flight behaviour are challenging to implement in laboratory environments, and they also struggle with identity tracking, particularly during occlusions. Here, we introduce FlightTrackAI, a robust convolutional neural network (CNN)-based tool for automatic mosquito flight tracking. FlightTrackAI employs CNN, a multi-object tracking algorithm, and interpolation to track flight behaviour. It automatically processes each video in the input folder without supervision and generates tracked videos with mosquito positions across the frames and trajectory graphs before and after interpolation. FlightTrackAI does not require a sophisticated setup to capture videos; it can perform excellently with videos recorded using standard laboratory cages. FlightTrackAI also offers filtering capabilities to eliminate short-lived objects such as reflections. Validation of FlightTrackAI demonstrated its excellent performance with an average accuracy of 99.9%. The percentage of correctly assigned identities after occlusions exceeded 91%. The data produced by FlightTrackAI can facilitate analysis of various flight-related behaviours, including flight distance and volume coverage during flights. This advancement can help to enhance our understanding of mosquito ecology and behaviour, thereby informing targeted strategies for vector control.

## 1. Introduction

Diseases transmitted by mosquitoes are medically significant due to their global distribution and further implications for public health [1]. Annually, malaria alone leads to approximately 230 million cases [2], notably affecting children under the age of 5 years [3]. Dengue virus is responsible for an estimated 390 million infections globally [4]. Beyond their impact on public health, mosquito-borne pathogens can also modulate various mosquito behaviours, such as flight, egg-laying, fertility and feeding [5,6]. Furthermore, recent studies indicate that viruses transmitted by vectors can infect and substantially affect the nervous systems of mosquitoes [7–9].

Understanding the behavioural characteristics of mosquitoes holds significance due to their role as crucial determinants in the transmission of pathogens and the epidemiology of mosquito-borne diseases. Attributes like fitness, host-seeking and host-feeding play pivotal roles in the severity of the diseases they spread. Examining the flight behaviour of mosquitoes becomes essential for evaluating their fitness, offering insights into how diverse mosquito populations adapt to different environments [10,11] and shedding light on the intricate relations between mosquitoes and the pathogens they carry [12,13]. Such detailed behavioural insights can inform targeted vector control strategies, enabling more precise interventions to alter key behaviours associated with pathogen transmission.

Traditionally, researchers have relied on manual observations to study the behaviours of mosquitoes. However, this method is resource-intensive and restricts the simultaneous monitoring of the number of mosquitoes. Additionally, some behavioural studies require continuous observations, leading to a laborious and time-consuming monitoring process. Various methodologies have recently been introduced to monitor mosquito flight behaviours. Khan *et al*. introduced a technique that involves the optimal placement of multiple pan-tilt-zoom (PTZ) infrared cameras within a three-dimensional virtual environment for the observation and tracking of mosquito trajectories [14]. Wilkinson *et al*. employed *in silico* simulations to monitor the flight activity of *Aedes albopictus* mosquitoes [15]. McMeniman *et al*. utilized the Track3D software and 210 frames per second (FPS) cameras to monitor active mosquitoes [16]. Straw *et al*. proposed simulating the three-dimensional tracking of flying animals using a network of cameras with high spatial resolution and frame rate [17]. Keller *et al*. developed the Photonic Fence (PF), a system that detects and tracks insects including mosquitoes using optical tracking and applies lethal doses of laser light to flying insects [18], which was further tested by Patt *et al*. in a large, field-scale set-up using machine vision, infrared light and lasers [19]. While advancements have been made in mosquito monitoring methods, their practical implementation in laboratory settings poses further challenges. Moreover, these approaches often suffer from limitations such as focusing only on active mosquitoes, the absence of individual mosquito identity while tracking and significant performance degradation in identification in the cases of occlusions.

Recently, artificial intelligence (AI) has played a significant role in revolutionizing visualization [20–23]. AI imitates human intelligence processes via various processes incorporated into a dynamic computing environment [24]. In the past, models based on AI have been used to examine different aspects of mosquitoes, including identifying breeding sites [25], counting mosquito eggs [26] and determining gender [27]. AI has also demonstrated its utility in assessing mosquito control [28].

To address the limitations of existing methods, we previously employed a convolutional neural network (CNN)-based approach for monitoring mosquito flight behaviour [29]. This method effectively tracked both resting and flying mosquitoes. However, its performance was less effective in occlusion situations due to its reliance on a basic L2-norm distance-based approach for assigning identities to tracked mosquitoes. Furthermore, manual post-processing steps, such as interpolation, were required. In this study, we introduce the FlightTrackAI, which utilizes CNN, a multi-object tracking algorithm, and cubic spline interpolation to track the flight behaviour of mosquitoes. FlightTrackAI does not require videos to be captured with a sophisticated set-up; it can perform excellently with videos recorded using standard laboratory cages. The tool takes a folder of videos as input, automatically processes each video without requiring supervision, and generates tracked videos with detailed mosquito locations and trajectory graphs before and after interpolation. By providing comprehensive data on mosquito behaviour patterns, FlightTrackAI has the potential to enhance vector control strategies and refine epidemiological models, leading to more precise and timely interventions.

## 2. Material and methods

### 2.1. Mosquito rearing

All experiments were conducted in accordance with biosafety level 3 guidelines at the insectary facility located at the CSIRO Australian Centre of Disease Preparedness. *Aedes aegypti* mosquitoes were raised under controlled conditions at a temperature of 27.0°C, maintaining a relative humidity of 70% and adhering to a 12 L : 12 D cycle. Adult mosquitoes were continuously supplied with a 10% sucrose solution for sustenance.

### 2.2. Experimental set-up

**Figure 1.**
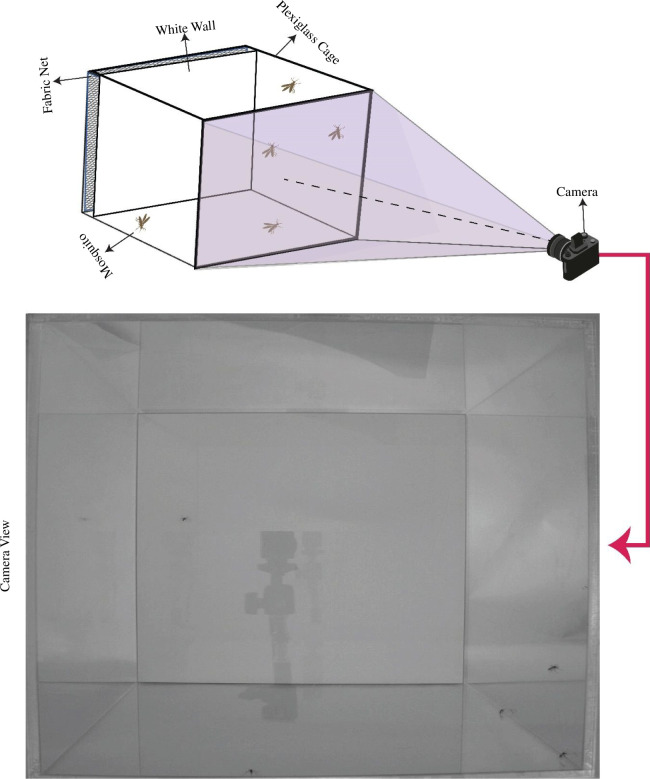
Experimental set-up.

A Plexiglas enclosure (25 cm × 25 cm × 40 cm) was used to isolate *A. aegypti* mosquitoes. This enclosure featured a movable white Plexiglas wall to eliminate background interference from standard netting sleeves. By positioning the white wall between the net and the recording area of the cage, we ensured that video footage was free from obstruction for accurate inspection. Video data collection was facilitated using infrared capture technology (Flea3, Point Grey Research, Canada) (figure 1).

### 2.3. Data collection

The recording was started two weeks post-emergence. The videos were recorded under 100% light conditions in the laboratory. The light pattern in the laboratory was: 0% light as ‘night’ for 8 h, 10% light for 1 h, 50% light for 1 h as ‘dawn’, 100% light as ‘day’ for 12 h and 50% light for 1 h, then 10% light for 1 h as ‘dusk’. Video data of 120 min duration were recorded at 60 FPS, with five mosquitoes inside the cage. Despite the Flea3 camera’s native resolution being 1280 pixels in width and 1024 pixels in height, adjustments were made to set the frame size at 1040 pixels in width and 1024 pixels in height. This configuration was tailored to match the dimensions of the enclosure and prevent the capture of extraneous background.

### 2.4. Data processing

The majority of flight-related behavioural analyses only focuses on individual mosquitoes' behaviour [30,31]. In this study, the recorded video was subdivided into smaller segments, with each segment corresponding to a single mosquito flight. In instances where multiple mosquitoes were in flight concurrently, individual trimming was applied to ensure that each segment captured the flight of a single mosquito. With a total of 52 flights, the recorded video data were segmented into 52 individual video segments using Digiarty [32].

### 2.5. Training, validation and test data

**Figure 2.**
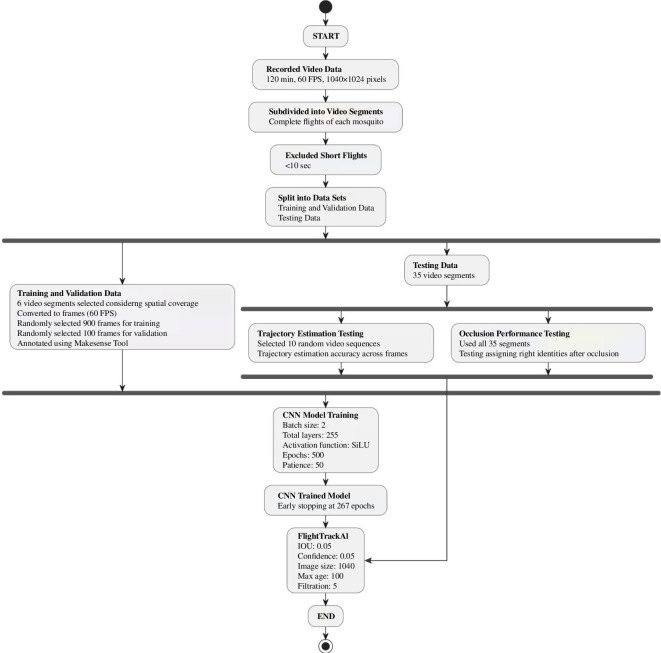
Workflow for data processing, model training and testing.

Out of the 52 flights, 11 were short in duration, lasting less than 10 s, and were consequently excluded from the dataset. For the customized training of the Yolov8 model from the remaining video segments, we opted for six randomly selected video segments (around 15% of the total number of remaining videos), each spanning durations between 31 and 99 s, to serve for training and validation datasets. While selecting these video segments, the spatial coverage of mosquitoes during the flight was considered, ensuring they covered most regions of the cage including edges and boundaries for comprehensive training. The remaining 35 segments, with durations ranging from 12 to 286 s, were reserved for performance testing. The average duration of the mosquito’s flight was 52.13 s. Therefore, 10 consecutive videos from average duration onwards, ranging from 54 to 131 s, were selected for trajectory testing, while all 35 videos were used for occlusion analysis. We converted training and validation videos into images at 60 FPS using the Python OpenCV library to prepare the data for training and validation. Out of these images, 900 were randomly chosen for training and 100 for validation. The CNN model underwent training for 500 epochs, but due to the early stopping patience set at 50, the training halted at 267 epochs, giving no observed improvement in performance and indicating that the best performance observed was at epoch number 217. An epoch represents a complete iteration over the entire training dataset during the training process. To annotate the training and validation data, we utilized the Makesense web tool, generating .text files [33] (figure 2).

### 2.6. Ground truth calculation

**Figure 3.**
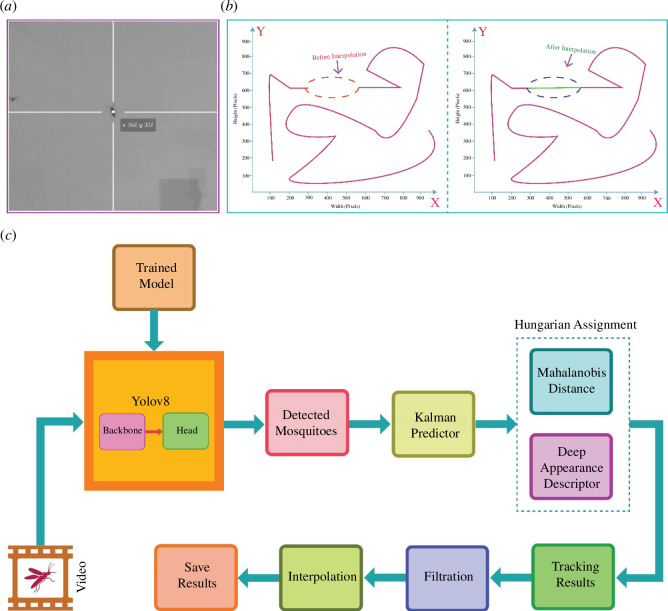
Methodologies. (*a*) Ground truth centroid calculation using the Makesense web tool. (*b*) Visualization of how interpolation fills missing data points. (*c*) FlightTrack AI’s methodology to estimate trajectories.

Mosquito ground truth positions were also validated through the Makesense web tool [33]. The tool allows the import of images, permitting the confirmation of object positions by navigating the cursor across the screen (figure 3*a*).

### 2.7. Trajectory estimation evaluation

Evaluating how well a tool performs its function is crucial in understanding its effectiveness. The FlightTrackAI tool’s trajectory evaluation performance is assessed by considering both mean absolute error (MAE) and accuracy. The accuracy metric evaluates whether the detected centroids meet the specified criteria, which considers both the position of the ground truth centroids and the tolerance allowed, while the MAE provides a detailed analysis of the variations between the detected centroids and the ground truth centroids, offering an insight into the precision of the FlightTrackAI tool’s performance. The MAE takes into account deviations along both the *x*- and *y*-axes in pixels, calculated using equation (2.1). The Python library Numpy is employed to compute MAE.


(2.1) $$MAE=\frac{1}{n}\sum_{f=1}^{n}|\left(x_{f\;}-{\hat{x}}_{f}\right)|+|\left(y_{f}-{\hat{y}}_{f}\right)|,$$

where $x_{f}$ is *x*-axis estimated position in pixels, ${\hat{x}}_{f}$ is *x*-axis ground truth position in pixels, $y_{f}$ is *y*-axis estimated position in pixels and ${\hat{y}}_{f}$ is *y*-axis ground truth position in pixels. The value of *f* represents the frame number, MAE is the mean absolute error across all the frames and *n* indicates the total number of frames.

In this context, accuracy refers to how closely the FlightTrackAI tool captures trajectory points compared to the trajectory points of the ground truth. A tolerance of six pixels is set for accuracy, indicating that estimated points within this range [absolute value on both horizontal (*x*-axis) and vertical axis (*y*-axis)] of the ground truth centroids are considered accurate (equation (2.2)). This choice is grounded in the understanding that mosquitoes are not single-pixel organisms, and given their varied positions, exact centroid estimation is challenging. Moreover, there are also chances of errors while calculating exact ground truth centroids manually, further emphasizing the need for a reasonable tolerance in the accuracy assessment. The six-pixel threshold is determined by randomly selecting 20 frames, taking the average of mosquito lengths in selected frames and dividing the average by two. This value corresponds to 0.57% of the *x*-axis total pixels and 0.58% of the *y*-axis total pixels.


(2.2) $$if\;\left\{ \begin{array}{l} |\left(x_{f}-{\hat{x}}_{f}\right)|<6\;\;\cap \;\;|\left(y_{f}-{\hat{y}}_{f}\right)|<6\;\;\;\;\;\;\;\;\;\;Accurate\\ \;\;\;\;\;\;\;\;\;\;\;\;\;\;\;\;\;\;\;\;\;\;\;\;\;\;\;\;\;\;\;\;\;else\;\;\;\;\;\;\;\;\;\;\;\;\;\;\;\;\;Inaccurate \end{array}\right.$$

### 2.8. Occlusion calculation

In our analysis, occlusions were determined using Python, specifically by computing the intersection over union (IoU) between the bounding boxes detected by the CNN for mosquitoes in the frames of videos. A threshold of 0.25 (25%) was chosen for IoU; if the IoU value between the detected bounding boxes of mosquitoes exceeded this threshold, the mosquitoes were considered occluded. This threshold, demonstrating partial overlap, was chosen to address the possibility that selecting a high threshold could result in overlapping mosquitoes being perceived as single mosquitoes before their calculation as occluded. Furthermore, occlusions were also manually verified by watching the videos. IoU was calculated using equation 2.3, given below.


(2.3) $$IoU=\frac{Area\;of\;intersection}{Area\;of\;union}$$

### 2.9. Computation and programming system

The FlightTrackAI was developed utilizing an AMD Ryzen 9 5900 HX processor integrated with Radeon Graphics (3.30 GHz), and 2x16 GB SO-DIMM DDR4-3200 RAM, operating within a Windows 11 Pro 64-bit environment. FlightTrackAI performs detection and tracking with the assistance of Python, Numpy, Scipy, Tensorflow and OpenCV.

### 2.10. Model architecture

FlightTrackAI uses YOLOv8 for object detection [34], Deep SORT for multi-object tracking [35] and cubic spline interpolation to fill in the missing data points. The YOLOv8 architecture employs distinct backbone and head sections to execute object detection tasks. The CSPDarknet53 serves as the backbone, utilizing convolutional layers to extract key features from the input image. The SPPF layer and the following convolution layers deal with features of different sizes, and the Upsample layers make the feature maps clearer. The C2F module combines important features and contextual data to improve detection accuracy. Finally, the head section utilizes a mix of convolutional and linear layers to convert complex features into output bounding boxes and classes. The model used 225 layers, including all convolutions, skip connections and other operations throughout the model. Advanced activation function SiLU (Swish) was used, improving gradient flow and feature expressiveness. This design reduces computational complexity, enhances gradient flow, improves feature reuse and maintains high accuracy while reducing model size.

Deep SORT performs multi-object tracking using the Kalman filter and the Hungarian algorithm equipped with deep association metrics. The Kalman filter is employed for predicting the state of objects, encompassing parameters like position, velocity and acceleration. This prediction is based on the last known state of the object, incorporating considerations for the dynamic motion of the object. Simultaneously, the Hungarian algorithm performs matching by evaluating a cost matrix, which considers both the Mahalanobis distance for motion consistency and the cosine distance for appearance similarity. Incorporating a deep association metric enables deep SORT to maintain tracking continuity even during short periods of occlusion. Then, FlightTrackAI employs cubic spline interpolation to smoothly fill in the missing data points, if there are any. This technique involves creating a smooth curve that passes through all the points in the dataset. Cubic spline interpolation seeks to create a smooth and uninterrupted curve at every data point using a cubic polynomial sequence (figure 3*b*). FlightTrackAI uses the Python library Scipy to perform interpolation. In the end, mosquito positions' data are saved in .xlsx format and before and after interpolation trajectories are generated using the Matplotlib Python library (figure 3*c*).

### 2.11. Mosquito flight tracking using FlightTrackAI

The FlightTrackAI’s user interface provides five adjustable input parameters for effective mosquito flight tracking. The model also requires a trained model, which can be selected by clicking on the ‘Select Model’ button. A pre-trained model is included with the tool. However, if custom training is desired, users can follow the straightforward instructions provided in [34] to train their own model. The adjustable input parameters include Image Size, Confidence, IoU, Max Age and Filtration. The Image Size parameter lets users set the maximum dimension of the input image, allowing adaptability across various resolutions. Considering the largest dimension of our experimental videos, its default value is set to 1040; it can be adjusted to any required value, and then FlightTrackAI will automatically re-adjust it to the nearest acceptable value, which should be a multiple of maximum stride 32, for compatibility with the CNN’s architecture. For example, in our case, it was automatically adjusted to 1056. The Confidence parameter filters out detections with confidence scores below the specified threshold, ensuring that only high-confidence detections contribute to the final results. The default value is set to a low value (0.05) to accommodate small sizes of mosquitoes. The next parameter is the IoU, which is critical for Non-Maximum Suppression (NMS) during the detection phase. It helps to eliminate redundant bounding box predictions. The default IoU threshold is set at 0.05, promoting a lenient evaluation of spatial overlap during the NMS step, which is particularly useful for detecting small and closely spaced objects.

Additionally, FlightTrackAI includes the Max Age parameter, determining how many frames a detection remains in the tracking system if undetected for a certain number of frames. This parameter is essential for accurate tracking over time, preventing the system from retaining outdated or incorrect information. The last parameter is the Filtration parameter, which is crucial as it helps to eliminate identities with short durations. This parameter is essential for excluding identities resulting from reflections on Plexiglas surfaces, ensuring more precise and clean final results. By default, its value is set to 5, meaning that if an identity appears for less than 5% of total frames, it will be removed from the final results.

**Figure 4.**
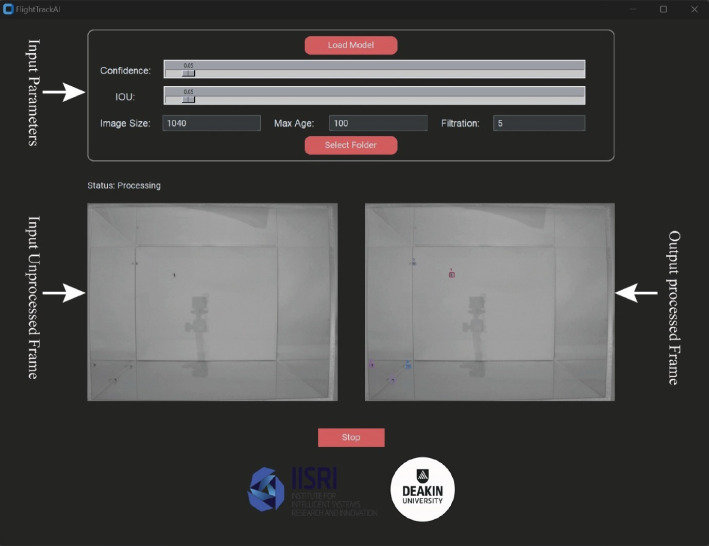
Graphical user interface of FlightTrackAI.

After setting these parameters, the input folder containing the videos can be selected by clicking on the ‘Select Folder’ button. Selecting the input folder will automatically start the processing. After processing each video, the FlightTrackAI generates a subfolder within the 'Results' directory located inside the input folder, named after the respective video in the input folder. This subfolder stores processed video and information about the positions of mosquito axes and the trajectories before and after interpolation. Besides saving the output results in the ‘Results’ folder, the FlightTrackAI will also show the unprocessed input and processed output frames in the user interface during processing. The FlightTrackAI allows users to halt the operation at any point by simply pressing the stop button at the bottom. Furthermore, it ensures that any partially processed video is automatically saved along with the information about the positions of mosquito axes and the trajectories before and after interpolation. The FlightTrackAI graphical user interface can be visualized in figure 4.

## 3. Results

**Figure 5.**
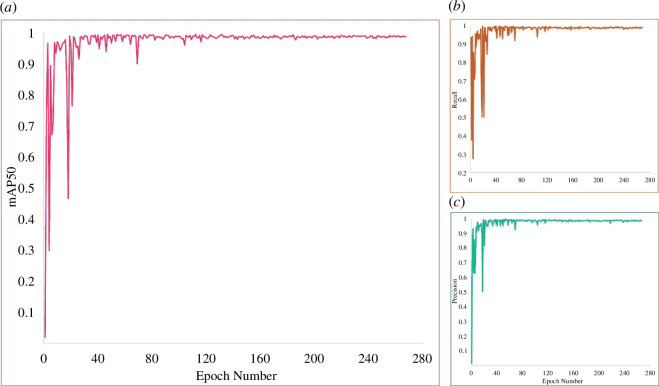
Training results. (*a*) mAP50, (*b*) recall and (*c*) precision.

The object detection model has demonstrated outstanding performance in detecting mosquitoes. The validation results exhibited high precision (0.986), recall (0.988) and mAP50 of 0.989, where mAP50 is the mean average precision calculated at an IoU threshold of 0.50. The precision, recall and mAP50 values across the training epochs are shown in figure 5*a–c*.

**Figure 6.**
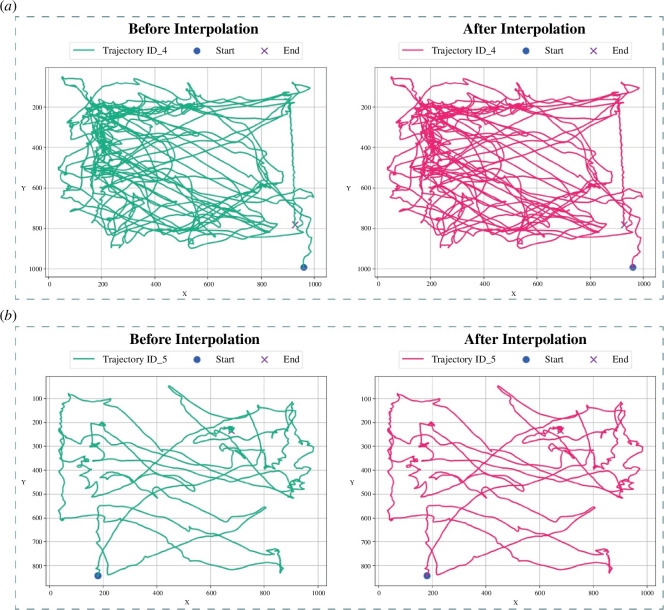
Results visualization. (*a,b*) Results before and after interpolation for two different flights.

Analysis of the FlightTrackAI’s performance on 10 test videos revealed that the object detector consistently identified mosquitoes in the majority of frames, resulting in a minimal percentage of interpolated data ranging from 0% to 0.67% across all videos, with an average of 0.20%. Additionally, the multi-object tracker exhibited exceptional capabilities, correctly assigning identities in 92 out of 101 occlusion instances, achieving a success rate exceeding 91% (table 1). Visual representations in figure 6*a,b* showcase trajectories of two distinct flights before and after interpolation, highlighting their nearly identical appearance due to the minimal amount of interpolated data. Processed videos can be visualized in the supplementary data.

**Table 1.** Test videos' CNN detection and interpolation results.

flight number	total frames	CNN detected	interpolated	interpolation percentage
**1**	3241	3221	20	0.61
**2**	3361	3361	0	0
**3**	3661	3661	0	0
**4**	3721	3696	25	0.67
**5**	3841	3818	23	0.59
**6**	4381	4374	7	0.15
**7**	4741	4738	3	0.06
**8**	4741	4734	7	0.14
**9**	5821	5821	0	0
**10**	7861	7852	9	0.01
**Total**	45 370	45 276	94	0.20

## 4. Trajectory estimation evaluation

**Figure 7.**
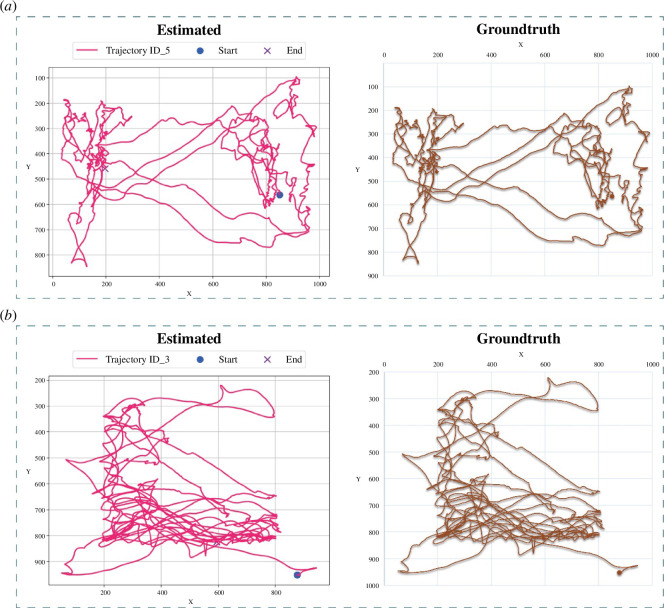
Trajectory evaluation results. (*a,b*) Estimated versus ground truth trajectories for two different flights.

FlightTrackAI’s trajectory estimation performance was validated by assessing its performance on 10 test videos using accuracy and MAE-encompassing error across both the *x*- and *y*-axes. The tool demonstrated a commendable accuracy, ranging from 99.89% to 100%, with an overall average of 99.98%. The MAE, encompassing both axes, varied between 0.20 and 0.27 pixels, yielding an average of 0.236 pixels (table 2). Figure 7 is displaying the estimated and ground truth trajectories of two different flights. Estimated and ground truth trajectories are looking almost identical in both charts, highlighting the tool’s outstanding performance.

**Table 2.** Trajectory estimation evaluation results.

flight number	total frames	incorrect detection frames	mean absolute error (pixels)	accuracy%
**1**	3241	3	0.25	99.91
**2**	3361	0	0.23	100
**3**	3661	0	0.24	100
**4**	3721	3	0.26	99.91
**5**	3841	4	0.27	99.89
**6**	4381	0	0.24	100
**7**	4741	0	0.22	100
**8**	4741	0	0.24	100
**9**	5821	0	0.21	100
**10**	7861	1	0.20	99.99
**total**	45 370	11	0.236	99.98

**Figure 8.**
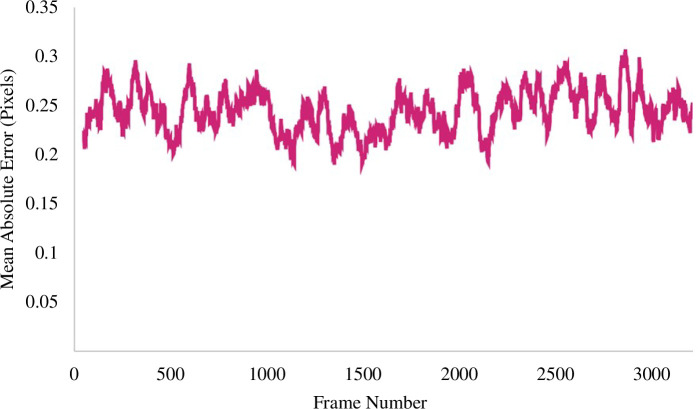
Mean absolute error across the frames.

Figure 8 illustrates the average of the first 3241 frames across all 10 videos, selected based on the minimum frame count of the smallest video. To facilitate a clear interpretation of the data, a moving average with a 50-frame period was applied, enhancing comprehension of the chart.

## 5. Discussion

The FlightTrackAI can facilitate a comprehensive analysis of mosquito flight behaviour, including studies on ecological interactions, the impacts of environmental changes and comparisons of behaviour before and after infection. Data generated from FlightTrackAI can be used to study various aspects of flight behaviour, such as diurnal and nocturnal flight activity, host-seeking behaviour, flight distance and velocity [36–39]. Additionally, synchronizing two cameras facilitates the capture of three-dimensional data [40], enabling FlightTrackAI to analyse three-dimensional flight parameters, such as the volume covered during flights, by processing the data from each camera individually. Moreover, considering the tool’s commendable performance in analysing the flight behaviour of mosquitoes, which are small in size and exhibit complex flight patterns, it demonstrates the potential for broader use in studying other organisms like flies, ticks and zebrafish.

The performance of FlightTrackAI was tested using five mosquitoes in a cage at a time, with a maximum of three mosquitoes flying simultaneously (electronic supplementary material, video SB). In previous work, we observed that using five mosquitoes in a cage allowed us to distinguish the flight traits, such as the volume covered during flight, of dengue-infected and non-infected mosquitoes [6]. While it is possible to increase the number of mosquitoes, doing so may impact the tool’s ability to assign correct identities after occlusion. Thus, balancing the number of mosquitoes with respect to the cage size is crucial for maintaining accurate tracking and identification performance.

Table 3 compares the tool’s performance with other available mosquito flight tracking methods, offering valuable insights into its effectiveness. While most methods did not offer accuracy metrics, those that did indicate that our proposed method achieves a superior accuracy rate of 99.98%. It is important to note that the testing conditions, such as experimental set-up, mosquito species, number of mosquitoes tracked and mosquito activity monitored, were different across the studies. For instance, Khan *et al*. used PTZ infrared cameras in a three-dimensional virtual environment to monitor mosquito colonies, employing a camera placement optimization algorithm to ensure full coverage and minimal occlusion [14]. Wilkinson *et al*. utilized *in silico* simulations to track *A. albopictus* flight activity in a controlled environment room, filming at 25 FPS [15]. McMeniman *et al*. employed the Track3D software with 210 FPS cameras for tracking *A. aegypti*, focusing on high-speed flight activities [16]. Keller *et al*. developed the PF, which uses optical tracking and laser application to target and eliminate *Anopheles stephensi* mosquitoes in flight [18]. These set-ups vary significantly in terms of complexity and the nature of mosquito activity they can capture. However, almost all these methods lack the ability to maintain the identities of mosquitoes during flights, do not have a graphical user interface, some require complex setups and some require extensive manual work.

**Table 3.** Comparing mosquito flight tracking methods.

year	tracking method	accuracy (%)	reference
**2011**	network of cameras with high spatial resolution and frame rate	-	[17]
**2012**	stereo vision techniques	-	[41]
**2014**	*in silico* simulations	>90	[15]
**2014**	track3D software and high-speed cameras	-	[16]
**2015**	Pan-Tilt-Zoom (PTZ) infrared cameras' placement within a three-dimensional virtual environment	-	[14]
**2020**	photonic fence		[18]
**2023**	convolutional neural network and interpolation	96	[29]
**2024**	photonic fence		[19]
**2024**	**proposed method**	**99.98**	this work

In comparison, FlightTrackAI offers several advantages. Apart from its excellent performance, the proposed approach features a user-friendly graphical interface. This interface simplifies the video processing workflow by enabling batch processing of videos within designated folders without constant supervision. Furthermore, the tool incorporates filtering capabilities to eliminate short-lived objects, such as reflections, from the final results, thereby enhancing the reliability of the outcomes. FlightTrackAI does not require a sophisticated set-up to capture videos; it can perform excellently with videos recorded using standard laboratory cages.

FlightTrackAI has demonstrated excellent performance in tracking mosquitoes from videos captured at 60 FPS, even with occlusions. However, its performance in assigning the correct identities may drop at lower video frame rates. Previous animal tracking studies have recommended frame rates above 25 FPS for animals such as mice, zebrafish and ants [42,43]. Considering mosquitoes' small sizes, fast speed and intricate flight patterns, it may be necessary to use videos with higher frame rates compared to frame rates required for mice, zebrafish and ants. Furthermore, significant gaps in data points resulting from background distortions and light reflections might impact interpolation performance. Therefore, this study suggests utilizing a clean background, as demonstrated by introducing a simple white wall in this work. Additionally, increasing the training data and utilizing anti-glare Plexiglas can also help to enhance performance by minimizing environmental distractions and optimizing the input quality for data processing. A few challenges observed during the experiments include ensuring that the size of the white wall is fit enough to prevent mosquitoes from crossing the wall and moving out of the camera’s view. Furthermore, the position of the cage is also critical; it should be located where light reflections have minimal impact on the camera view.

In real field settings, challenges such as varying environmental factors, including changing light conditions, complex backgrounds and natural obstacles, might affect the performance of FlightTrackAI. However, the robustness of the CNN and multi-object tracking algorithms employed by FlightTrackAI can maintain high accuracy and reliability, making it a valuable tool even under field conditions. Additionally, the presence of non-target organisms could also impact the performance of FlightTrackAI. Nevertheless, CNNs can show excellent performance in distinguishing mosquitoes from non-target organisms, as previously CNNs have been used to classify different small insects [44] and different species of mosquitoes [45] and have shown high accuracy. By continuously refining the training data and incorporating diverse field scenarios, we can further enhance its effectiveness in practical applications.

The integration of advanced monitoring tools like FlightTrackAI into mosquito research holds a significant promise for enhancing vector control strategies and refining epidemiological models. FlightTrackAI has the potential to improve the effectiveness of vector control programs by providing comprehensive data on mosquito behaviour patterns, enabling more precise and timely interventions. By accurately tracking mosquito flight, FlightTrackAI can enhance the predictive accuracy of models forecasting the spread of mosquito-borne diseases [46], leading to more precise and dynamic epidemiological models. Data generated by FlightTrackAI can also be used to monitor changes in different parameters such as velocity, flight angle, proximity to objects and flight patterns when a mosquito approaches objects such as human hands, mosquito traps or repellents. These insights can reveal crucial details about mosquito interactions with their environment and responses to control measures. For example, understanding how mosquitoes change their behaviour in response to a repellent can help in assessing the repellent’s effectiveness. Similarly, tracking mosquito interactions with traps can optimize trap placement and design. This capability can help assess the effectiveness of various control measures. With these insights, public health officials can develop more informed vector control strategies that are better adapted to mosquito behaviour patterns. Ultimately, these advancements not only advance our understanding of mosquito behaviour but also improve our capacity to combat vector-borne diseases, potentially reducing disease transmission and enhancing public health outcomes.

## 6. Conclusions

In conclusion, the development of FlightTrackAI utilizing CNN, multi-object tracking algorithm and cubic spline interpolation represents a significant advancement in understanding the flight behaviour of mosquitoes. This tool enables accurate analysis of mosquito flight behaviour through automatic video data processing and robust tracking capabilities. The validation results of FlightTrackAI demonstrate its high accuracy (99.98%) and lower average MAE (0.236 pixels), making it a valuable asset for researchers looking into exploring the intricate pathogen–mosquito relationship.

The FlightTrackAI sets the stage for creating automated mosquito behaviour monitoring systems which are crucial for assessing the fitness of insects, particularly in the context of infection or genetic modification studies. Furthermore, such advancements hold immense potential for advancing vector-borne disease modelling and the development of innovative trapping methods.

Future work could expand the AI’s capabilities to automatically track feeding-related behaviours, such as feeding instances and feeding durations, thereby providing a more comprehensive understanding of the pathogen–mosquito relationship. Overall, this research paves the way for further exploration and application of automated tracking technologies in entomology and beyond.

## Data Availability

Supplementary videos, videos for testing and FlightTrackAI graphical user interface .exe file and installation instructions are available at OSF and can be accessed using the following link [47] Supplementary material is available online [48].
